# Crystal structure of the putative cell-wall lipoglycan biosynthesis protein LmcA from *Mycobacterium smegmatis*

**DOI:** 10.1107/S2059798322001772

**Published:** 2022-03-11

**Authors:** Onisha Patel, Rajini Brammananth, Weiwen Dai, Santosh Panjikar, Ross L. Coppel, Isabelle S. Lucet, Paul K. Crellin

**Affiliations:** a The Walter and Eliza Hall Institute of Medical Research, Parkville, Victoria 3052, Australia; b Infection and Immunity Program, Monash Biomedicine Discovery Institute and Department of Microbiology, Monash University, Victoria 3800, Australia; c Australian Synchrotron, Clayton, Victoria 3168, Australia; d Department of Molecular Biology and Biochemistry, Monash University, Victoria 3800, Australia

**Keywords:** *Mycobacterium tuberculosis*, *Mycobacterium smegmatis*, lipomannan, lipoarabinomannan, MSMEG_0317

## Abstract

The bacterial genus *Mycobacterium* includes important pathogens, most notably *M. tuberculosis*, which infects one-quarter of the entire human population, resulting in around 1.4 million deaths from tuberculosis each year. Myco­bacteria, and the closely related corynebacteria, synthesize a class of abundant glycolipids, the phosphatidyl-*myo*-inositol mannosides (PIMs). PIMs serve as membrane anchors for hyperglycosylated species, lipomannan (LM) and lipoarabinomannan (LAM), which are surface-exposed and modulate the host immune response. Previously, in studies using the model species *Coryne­bacterium glutamicum*, NCgl2760 was identified as a novel membrane protein that is required for the synthesis of full-length LM and LAM. Here, the first crystal structure of its ortholog in *Mycobacterium smegmatis*, MSMEG_0317, is reported at 1.8 Å resolution. The structure revealed an elongated β-barrel fold enclosing two distinct cavities and one α-helix extending away from the β-barrel core, resembling a ‘cone with a flake’ arrangement. Through xenon derivatiz­ation and structural comparison with *AlphaFold*2-derived predictions of the *M. tuberculosis* homolog Rv0227c, structural elements were identified that may undergo conformational changes to switch from ‘closed’ to ‘open’ conformations, allowing cavity access. An *AlphaFold*2-derived NCgl2760 model predicted a smaller β-barrel core with an enclosed central cavity, suggesting that all three proteins, which were collectively termed LmcA, may have a common mechanism of ligand binding through these cavities. These findings provide new structural insights into the biosynthetic pathway for a family of surface lipoglycans with important roles in mycobacterial pathogenesis.

## Abstract 2

The first crystal structure of the putative cell-wall biosynthesis protein LmcA from *Mycobacterium smegmatis* is reported at 1.8 Å resolution. The structure revealed an elongated β-barrel fold enclosing two distinct cavities, indicating a possible lipid-binding function in lipomannan/lipoarabinomannan biosynthesis.

## 1. Introduction

Bacteria of the suborder Corynebacterineae include important human pathogens such as *Mycobacterium tuberculosis*, *M. leprae* and *Corynebacterium diphtheriae*, and nonpathogenic species such as *M. smegmatis* and *C. glutamicum*, which serve as useful experimental models. *M. tuberculosis* infects around one-quarter of the entire human population and causes approximately 1.4 million deaths annually, making it one of the top ten causes of death worldwide (World Health Organization, 2020 ▸). A key virulence factor and validated drug target is the unusually hydrophobic, multilayered cell wall of these bacteria, which comprises a diverse variety of lipids with structural roles as well as important functions in interactions with the human host (Brennan & Nikaido, 1995 ▸; Jankute *et al.*, 2015 ▸).

One group of abundant glycolipids synthesized by all mycobacteria and corynebacteria are the phosphatidyl-*myo*-inositol mannosides (PIMs). The PIMs also serve as membrane anchors for hyperglycosylated species: lipomannan (LM) and lipoarabinomannan (LAM). These complex surface lipoglycans are essential for the viability and *in vivo* survival of pathogenic mycobacterial species due to their capacity to modulate host immune responses during infection (Chatterjee & Khoo, 1998 ▸; Maeda *et al.*, 2003 ▸; Mishra, Driessen *et al.*, 2011 ▸; Nigou *et al.*, 2002 ▸; Schlesinger *et al.*, 1994 ▸; Strohmeier & Fenton, 1999 ▸; Vercellone *et al.*, 1998 ▸). While many enzymatic steps of the PIM/LM/LAM biosynthetic pathway have been defined, generally through studies using *C. glutamicum* or *M. smegmatis* as a model, the mechanisms by which the pathway is regulated, how the various proteins cooperate to synthesize these lipoglycans and how the intermediates are transported through the cell-wall layers remain poorly understood.

**Figure 1:**
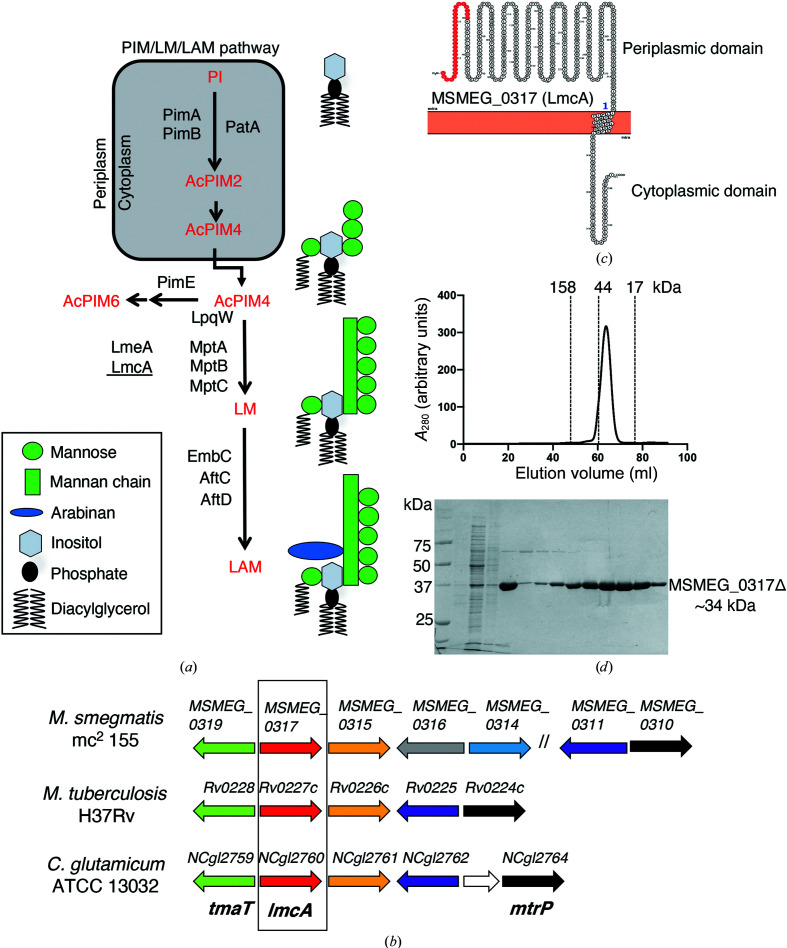
(*a*) The PIM/LM/LAM biosynthetic pathway of mycobacteria. Early steps of PIM synthesis are performed by the cytoplasmic enzymes PimA (Korduláková *et al.*, 2002 ▸), PimB (Guerin *et al.*, 2009 ▸; Lea-Smith *et al.*, 2008 ▸) and PatA (Korduláková *et al.*, 2003 ▸) to produce AcPIM2 from phosphatidylinositol (PI). Further mannosylation yields AcPIM4, which is transported to the periplasm and can be processed by the mannosyltransferase PimE (Morita *et al.*, 2006 ▸) to form AcPIM6, an end product, or channelled into a parallel pathway for LM and LAM synthesis by the lipoprotein LpqW (Crellin *et al.*, 2008 ▸; Kovacevic *et al.*, 2006 ▸; Marland *et al.*, 2006 ▸). LM/LAM synthesis is catalysed by the PPM-dependent mannosyltransferases MptB, MptA and MptC (Kaur *et al.*, 2006 ▸, 2007 ▸; Mishra *et al.*, 2007 ▸, 2008 ▸; Mishra, Krumbach *et al.*, 2011 ▸). A phospholipid-binding protein, LmeA (Rahlwes *et al.*, 2017 ▸), is involved in maintaining MptA under stress conditions (Rahlwes *et al.*, 2020 ▸). The focus of the current study, LmcA (underlined), also functions at the MptA step in *C. glutamicum* (Cashmore *et al.*, 2017 ▸). (*b*) The *MSMEG_0317* genetic locus. The *MSMEG_0317* gene is encoded within a locus that is highly conserved in Corynebacterineae. Likely orthologous genes in the three species are shown using the same colour. Previously studied genes are *tmaT* (Yamaryo-Botte *et al.*, 2015 ▸) and *mtrP* (Rainczuk *et al.*, 2020 ▸), both with roles in cell-wall mycolic acid transport, and the LM/LAM biosynthesis gene *NCgl2760* (Cashmore *et al.*, 2017 ▸), while the remaining genes are uncharacterized. The focus of the current study is boxed. (*c*) Predicted membrane topology of MSMEG_0317. Following cleavage of the putative signal peptide (red), the mature protein is proposed to comprise a large periplasmic N-­terminal domain, a single transmembrane domain and a small cytoplasmic tail. (*d*) The elution profile of MSMEG_0317Δ on a HiLoad 16/60 Superdex 75 gel-filtration column suggesting a monomeric protein (top) and SDS–PAGE analysis of the eluted MSMEG_0317Δ (∼34 kDa) (bottom). The molecular-weight markers used for calibration are bovine γ-globulin (158 kDa), chicken ovalbumin (44 kDa) and equine myoglobin (17 kDa). See also Supplementary Fig. S1.

Early steps in the pathway involve the mannosylation of PIM intermediates by GDP-mannose-dependent enzymes on the cytoplasmic face of the inner cell membrane, followed by transport to the periplasm (Fig. 1 ▸ *a*). Here, they can be converted to mature PIM end products or processed by a series of polyprenyl phosphomannoase (ppMan)-dependent mannosyltransferases that elongate the mannan backbone and add mannose side chains (MptA, MptB and MptC) to form LM (Kaur *et al.*, 2006 ▸; Mishra *et al.*, 2007 ▸, 2008 ▸; Mishra, Krumbach *et al.*, 2011 ▸). Other proteins [LpqW (Kovacevic *et al.*, 2006 ▸) and LmeA (Rahlwes *et al.*, 2020 ▸)] have additional roles in the reactions that synthesize LMs as regulators or possibly through lipid-binding activities. Finally, the addition of branched Ara*f* residues to LM by multiple arabinosyltransferases (AftC, AftD and EmbC) yields LAM (Alderwick *et al.*, 2011 ▸; Birch *et al.*, 2008 ▸; Mishra, Driessen *et al.*, 2011 ▸; Seidel *et al.*, 2007 ▸; Shi *et al.*, 2006 ▸; Skovierová *et al.*, 2009 ▸).

Previously, we identified a new membrane protein, conserved in Corynebacterineae, that is required for synthesis of full-length LM and LAM (Cashmore *et al.*, 2017 ▸). Deletion of the *NCgl2760* gene in *C. glutamicum*, a useful model organism for the study of cell-wall synthesis in Corynebacterineae, resulted in a complete loss of mature LM/LAM and the appearance of a novel truncated LM (t-LM). Lipid structural studies indicated that the Δ*NCgl2760* t-LM comprised a series of short LM species containing a truncated α(1–6)-linked mannose backbone with greatly reduced α(1–2) mannose side chains. These t-LM species were structurally similar to those of a *C. glutamicum* mutant lacking the MptA mannosyltransferase that extends the α(1–6) mannan backbone of LM intermediates (Mishra *et al.*, 2007 ▸), indicating that both proteins may act at a similar point in the pathway for LM (Cashmore *et al.*, 2017 ▸; Fig. 1 ▸ *a*). *C. glutamicum* NCgl2760 has putative orthologs in *M. smegmatis* (MSMEG_0317) and *M. tuberculosis* (Rv0227c), both of which are encoded by essential genes (Cashmore *et al.*, 2017 ▸; Griffin *et al.*, 2011 ▸; Sassetti *et al.*, 2003 ▸). Rv0227c has been localized to the bacterial surface and implicated in host cell entry by *M. tuberculosis* (Rodríguez *et al.*, 2012 ▸), but is otherwise unstudied.

NCgl2760, MSMEG_0317 and Rv0227c, which we collectively term LmcA, lack significant amino-acid sequence similarity to other proteins, making their function difficult to predict. To gain structural insight into the LmcA family, here we report the first crystal structure of the major domain of *M. smegmatis* LmcA at 1.8 Å resolution. Our crystal structure reveals an elongated β-barrel fold enclosing two distinct cavities. Xenon derivatization of the crystal structure further identified structural elements within the β-barrel that undergo conformational flexibility that allows cavity access. The *AlphaFold2*-derived *M. tuberculosis* Rv0227c model revealed an identical elongated β-barrel fold, consistent with our experimentally derived crystal structures, highlighting the accuracy of *AlphaFold*2-based predictions. While the *AlphaFold*2-modelled structure of the *C. glutamicum* ortholog NCgl2760 predicts a much smaller β-barrel fold, the most striking feature common to all three LmcA proteins is an enclosed central cavity, suggesting a common mechanism of ligand binding.

## 2. Materials and methods

### 2.1. Cloning, expression and purification of MSMEG_0317

For protein expression in *M. smegmatis*, a DNA sequence encoding the periplasmic domain of MSMEG_0317 was PCR-amplified using the primers MSMEG_0317pJAM-F (5′-CCAGGATCCACCTACACCAAGGGCAAG-3′) and MSMEG_0317pJAM-R (5′-CCTCTAGACCGTGTCCACAGCGCGATG-3′) and then cloned into the acetamide-inducible expression vector pJAM2 (Triccas *et al.*, 1998 ▸) using BamHI and XbaI (underlined), creating plasmid pPKC364. Following introduction into *M. smegmatis* mc^2^ 155 by electroporation, a single colony was inoculated into 10 ml Middlebrook 7H9 medium supplemented with ADS [5%(*w*/*v*) BSA, 0.85%(*w*/*v*) NaCl, 2%(*w*/*v*) glucose], 2 µl kanamycin (100 mg ml^−1^) and 0.5%(*v*/*v*) Tween-80 and cultured for three days at 37°C. This starter culture was diluted 1:20 into 2 l M63 minimal medium supplemented with 1 m*M* MgSO_4_, 0.5%(*v*/*v*) Tween-80, 0.2%(*w*/*v*) succinate and 2%(*w*/*v*) acetamide and grown for three days at 37°C. The cells were harvested by centrifugation at 3000*g* for 20 min, washed in PBS and the pellets were stored at −80°C.

The cells were resuspended in lysis buffer consisting of 20 m*M* Tris pH 7.5, 500 m*M* NaCl, 10%(*v*/*v*) glycerol, 5 m*M* imidazole, 0.1% Thesit supplemented with cOmplete EDTA-free Protease Inhibitor Cocktail (Roche) and lysed by sonication. The supernatant was clarified by centrifugation at 45 000*g* and 4°C for 30 min, filtered and loaded onto 1 ml Ni–NTA resin (Roche). After extensive washes with wash buffer [20 m*M* Tris pH 7.5, 500 *M* NaCl, 10%(*v*/*v*) glycerol, 5 m*M* imidazole], the protein was eluted in wash buffer supplemented with 150 m*M* imidazole. MSMEG_0317Δ-containing fractions were subjected to size-exclusion chromatography (SEC; Superdex 75 16/600, Cytiva) in SEC buffer [20 m*M* Tris pH 7.5, 200 m*M* NaCl, 5%(*v*/*v*) glycerol]. Fractions containing MSMEG_0317Δ protein were pooled and further purified by anion-exchange chromatography. MSMEG_0317Δ was diluted with buffer *A* [20 m*M* Tris pH 7.5, 5%(*v*/*v*) glycerol] and loaded onto a MonoQ 1/10 GL column (Cytiva) pre-equilibrated in buffer *A*. MSMEG_0317Δ was eluted with a gradient of buffer *B* [20 m*M* Tris pH 7.5, 1 *M* NaCl, 5%(*v*/*v*) glycerol] over 15 column volumes. MSMEG_0317Δ-containing fractions were pooled, concentrated to 5 mg ml^−1^ and flash-frozen for storage at −80°C.

### 2.2. Crystallization, data collection and structural determination

Poor-quality crystals of MSMEG_0317Δ were initially obtained through a random screen conducted at the C3 CSIRO facility in 2.7 *M* ammonium sulfate, 50 m*M* Tris pH 8.5. The crystals were then optimized by repeated rounds of microseeding and buffer optimization using the vapour-diffusion method. The best crystals were obtained by mixing 0.5 µl protein solution at 10 mg ml^−1^ with 0.5 µl reservoir solution consisting of 2.2 *M* ammonium sulfate, 50 m*M* Tris pH 7.0 in the presence of microseeds. Crystals were flash-cooled in liquid nitrogen in reservoir solution supplemented with 10%(*v*/*v*) glycerol. For iodide phasing, crystals were soaked in 0.25–0.5 *M* potassium iodide solution prior to cooling.

X-ray diffraction data for native MSMEG_0317Δ and iodide-derived MSMEG_0317Δ crystals were collected on the MX2 and MX1 beamlines at the Australian Synchrotron (Aragão *et al.*, 2018 ▸; Cowieson *et al.*, 2015 ▸), respectively. The native data were collected to 2.0 Å resolution at a wavelength of 0.9537 Å (referred to as MSMEG_0317Δ old native data in Table 1 ▸). SAD data were collected at a wavelength of 1.4586 Å from iodide-derived MSMEG_0317Δ crystals (referred to as MSMEG_0317Δ-KI in Table 1 ▸). Two data sets were collected from the iodide-soaked crystal at the same position of the crystal, but with an offset of 0.25° in oscillation range for the second data set. All diffraction data were processed using *XDS* (Kabsch, 2010 ▸) in space group *P*1 and the iodide data sets were merged in *AIMLESS* within the *CCP*4 suite (Evans & Murshudov, 2013 ▸; Winn *et al.*, 2011 ▸). Automated experimental phasing was carried out using the single-wavelength anomalous diffraction (SAD) phasing protocol of *Auto-Rickshaw* (Panjikar *et al.*, 2005 ▸, 2009 ▸). The input diffraction data were prepared and converted for use in *Auto-Rickshaw* using programs from the *CCP*4 suite (Winn *et al.*, 2011 ▸). 35 iodine sites with partial occupancy were bound to the protein. Further native data were run through the MR protocol of *Auto-Rickshaw* and were refined with *REFMAC* (Murshudov *et al.*, 2011 ▸). The resultant model contained 95% of the total residues. Subsequently, we collected a new native data at 1.8 Å resolution which replaced the original 2.0 Å resolution data set (referred to as MSMEG_0317Δ new native data in Table 1 ▸). The *R*_free_ set was copied from the original 2.0 Å resolution data and the model was further improved using manual model building in *Coot* (Emsley *et al.*, 2010 ▸) and refinement in *BUSTER* (Bricogne *et al.*, 2017 ▸). The final refined model has 98% of residues in the favoured region and 2% in allowed regions. A xenon pressure cell (Hampton Research) available at the Australian Synchrotron was used to pressurize MSMEG_0317Δ crystals with xenon before cryocooling following previously described protocols (Panjikar & Tucker, 2002 ▸). An MSMEG_0317Δ crystal in the loop was lowered into the xenon chamber and kept moist by placing ∼500 µl of the crystallization well solution (2.2 *M* ammonium sulfate, 50 m*M* Tris pH 7.0) at the bottom of the chamber. The chamber was held with 20 bar of xenon gas for 1 min and the gas was then released slowly. Soon afterwards, the looped crystal was plunge-cooled in liquid nitrogen. Data from the xenon-pressurized MSMEG_0317Δ crystal were collected at a wavelength of 0.9537 Å on the MX2 beamline of the Australian Synchrotron (referred to as MSMEG_0317Δ-Xe in Table 1 ▸). Two data sets were collected from different positions of the same crystal and processed using *XDS* in space group *P*1, followed by merging and scaling using *AIMLESS*. The crystal diffracted to 1.8 Å resolution, allowing xenon binding sites to be located unambiguously. The MSMEG_0317Δ-Xe structure was solved by the molecular-replacement method using *Phaser* (McCoy *et al.*, 2007 ▸) in the *CCP*4 suite, using the wild-type structure as a model, followed by model building and refinement in *Coot* and *BUSTER*, respectively. All structures were validated using *MolProbity* (Williams *et al.*, 2018 ▸). All molecular-graphics representations were created using *PyMOL* (version 2.3.4; Schrödinger). The topology diagram was generated using *Pro-origami* (Stivala *et al.*, 2011 ▸). *CASTp* was used for cavity analysis (Tian *et al.*, 2018 ▸). X-ray diffraction data-collection and refinement statistics are reported in Table 1 ▸. Coordinates and structure factors for native MSMEG_0317Δ and MSMEG_0317Δ-Xe have been deposited in the Protein Data Bank (PDB) with accession codes 7n3v and 7shw, respectively.

### 2.3. Sequence comparisons

Amino-acid and gene sequences were obtained from UniProt (http://uniprot.org; UniProt Consortium, 2021 ▸). Pairwise sequence alignment was carried out using the tools available from EMBL–EBI (http://www.ebi.ac.uk/Tools/psa/emboss_needle; Madeira *et al.*, 2019 ▸). *Clustal Omega* (Sievers *et al.*, 2011 ▸) from the same web server was used to generate multiple sequence alignments. High-resolution figures for the sequence alignment were prepared using *ESPript* (http://espript.ibcp.fr; Robert & Gouet, 2014 ▸). The sequence was checked for the presence of intrinsically disordered regions using the *IUPred* web server (Dosztányi *et al.*, 2005 ▸).

### 2.4. Modelling of *M. smegmatis* MSMEG_0317, *M. tuberculosis* Rv0227c and *C. glutamicum* NCgl2760

*M. tuberculosis* Rv0227c was predicted using the online AlphaFold Protein Structure Database developed by DeepMind and EMBL–EBI, and MSMEG_0317 and *C. glutamicum* NCgl2760 were predicted using AlphaFold Colab (AlphaFold2advanced.ipynb) and further confirmed using the full open-source *AlphaFold* package (Jumper *et al.*, 2021 ▸; Tunyasuvunakool *et al.*, 2021 ▸).

## 3. Results and discussion

### 3.1. LmcA is a putative membrane protein that is well conserved among mycobacteria and corynebacteria

Our previous studies on *C. glutamicum* LmcA (NCgl2760) provided strong evidence for a role in the formation of full-length LM and LAM (Cashmore *et al.*, 2017 ▸). NCgl2760 is encoded by a genetic locus that is well conserved in the Corynebacterineae suborder (Fig. 1 ▸ *b*) and is dedicated to cell-wall synthesis (Rainczuk *et al.*, 2020 ▸; Yamaryo-Botte *et al.*, 2015 ▸). In sequence-similarity searches, MSMEG_0317 was the best match for NCgl2760 in the *M. smegmatis* genome, with the proteins sharing 24% amino-acid sequence identity (Supplementary Fig. S1). This, combined with the genome synteny across multiple species, suggests that the proteins are orthologs. MSMEG_0317 and *M. tuberculosis* Rv0227c display higher identity (65%; Supplementary Fig. S1), as expected for proteins from species belonging to the same genus.

### 3.2. Expression, purification and structural determination

MSMEG_0317 is predicted to contain a signal peptide, a large periplasmic domain and a single transmembrane domain (residues 324–349) located towards the C-terminal end (Fig. 1 ▸ *c*). To gain insight into the structure of MSMEG_0317, we focused on the periplasmic domain and produced a truncated form that lacks the predicted signal peptide and transmembrane domain, referred to as MSMEG_0317Δ (residues 30–323), using a *M. smegmatis* expression system (Triccas *et al.*, 1998 ▸). Deletion of both the signal peptide and the transmembrane domain yielded a stable soluble form (Fig. 1 ▸ *d*). MSMEG_0317Δ eluted as a monomer on size-exclusion chromatography and yielded diffraction-quality crystals after several rounds of seeding (MSMEG_0317Δ old and new native data; Table 1 ▸). As structural homologs of MSMEG_0317 have not previously been characterized, we next soaked the native crystals with varying concentrations of halide ions, such as bromide and iodide. Crystals were able to tolerate 0.25 *M* potassium iodide without losing their crystalline order, allowing the collection of a SAD data set (MSMEG_0317Δ-KI; Table 1 ▸). The crystal structure of MSMEG_0317Δ was solved to 2.4 Å resolution and refined against the native data set using *Auto-Rickshaw* followed by refinement in *REFMAC*5, resulting in an almost complete model in space group *P*1 with two molecules in the asymmetric unit (Table 1 ▸, Supplementary Fig. S2).

### 3.3. MSMEG_0317Δ adopts an elongated β-barrel fold

**Figure 2:**
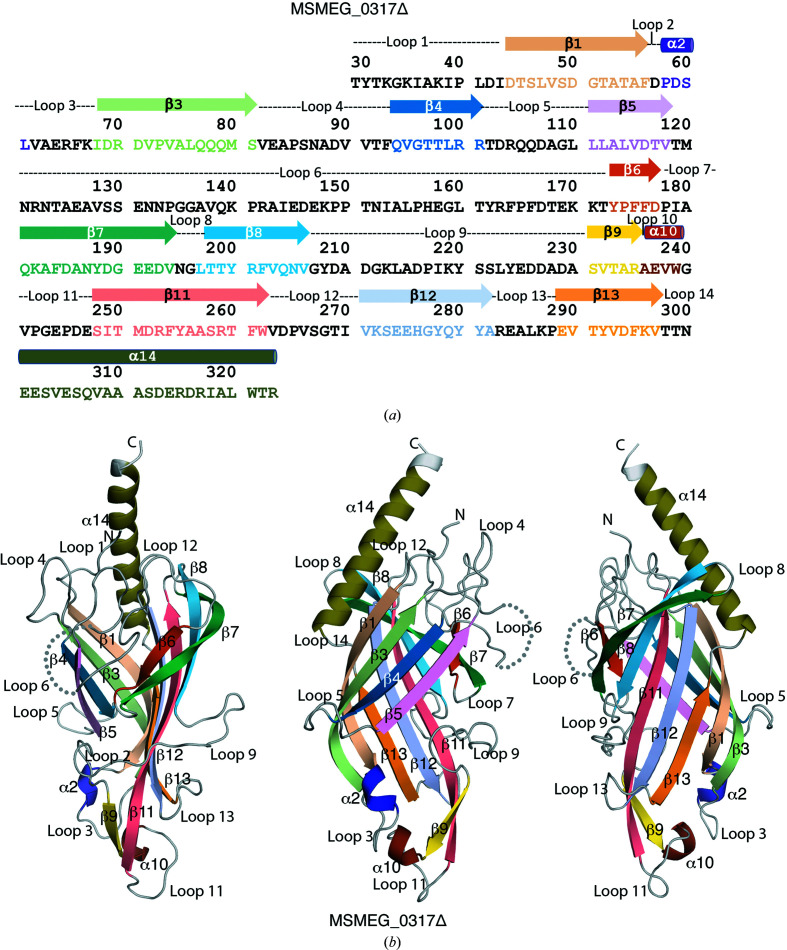
The amino-acid sequence and the crystal structure of the periplasmic domain of MSMEG_0317Δ. (*a*) The sequence of MSMEG_0317Δ showing secondary-structure elements derived from the crystal structure of MSMEG_0317Δ. (*b*) The crystal structure of MSMEG_0317Δ in different views. The secondary-structure elements are colour-coded. The disordered loop 6 is shown by dotted lines. See also Supplementary Figs. S2–S4.

MSMEG_0317Δ adopts an elongated β-barrel core composed of 11 antiparallel β-strands with two α-turns and one α-helix extending away from the core (Fig. 2 ▸, Supplementary Fig. S3). The N-terminal region folds back and interacts with the C-terminal α-helix located just before the transmembrane domain to form a closed structure that resembles the shape of a ‘cone with a flake’, with the cone being the β-barrel core and the extended α-helix being the flake. All the loops connecting the β-strands and α-turns are ordered except for residues 129–154 within loop 6, which connects β5 and β6 (Fig. 2 ▸ *b*). The wall of the β-barrel core is formed by two sets of antiparallel flat or twisted β-strands (Fig. 2 ▸ *b*). The first set of antiparallel β-strands is comprised of β1, β3, β4 and β5, which form one side of the β-barrel wall, and the second is comprised of β6, β7, β8, β11, β12 and β13, which form the opposite wall. Of these, β-strands β1, β3, β4, β6, β7 and β12 adopt twisted conformations to various degrees due to the presence of a glycine or a proline (Fig. 2 ▸ *b*). Each β-strand is connected to the subsequent β-strand through hydrogen-bond interactions, except for β8 and β9, which do not interact with each other directly but instead interact with β11 (Fig. 2 ▸, Supplementary Figs. S3 and S4*a* ). As the first strand β1 interacts with the last strand β13, the MSMEG_0317Δ fold resembles a closed toroidal β-barrel. The narrow base of the β-barrel core is occupied by α2, α10, β9 and the end of β11. As expected, the overall electrostatic potential of the MSMEG_0317Δ surface reveals a net positive charge near to the transmembrane domain attributed to the presence of Lys38 of loop 1, Arg122 of loop 6 and Arg317 of α14 (Supplementary Fig. S4*b* ). Interestingly, the surface electrostatics of residues on the surface of α14, β1, β3, β4 and β5 show an overall negative charge compared with surface residues in β8, β11, β12 and β13, suggesting that these may represent distinct surface interactions to accommodate the binding of interacting partners within the LM/LAM pathway.

### 3.4. The structure of MSMEG_0317Δ reveals two enclosed cavities

**Figure 3:**
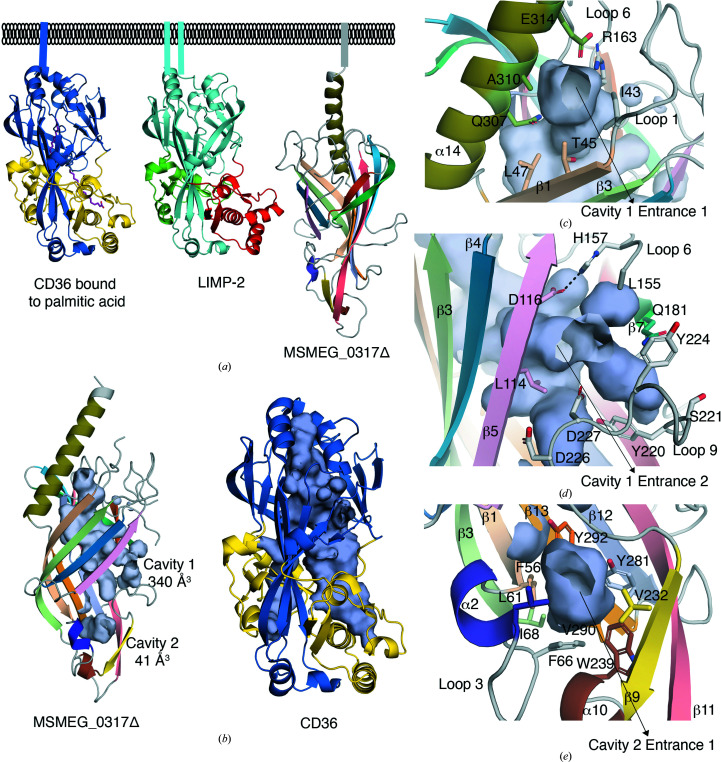
Structural homology and surface representation of the enclosed cavities in MSMEG_0317Δ. (*a*) Comparison of the MSMEG_0317Δ fold with the CD36 superfamily of scavenger receptor proteins, including the human lysosomal integral membrane protein 2 (LIMP-2) and CD36, a fatty-acid transporter, which show an extended asymmetric β-barrel core. (*b*) Comparison of the MSMEG_0317Δ enclosed cavities with the CD36 cavity which binds palmitic acid. (*c*) Close-up of MSMEG_0317Δ cavity 1 entrance 1. (*d*) Close-up of MSMEG_0317Δ cavity 1 entrance 2. (*e*) Close-up of MSMEG_0317Δ cavity 2. Hydrogen-bond and salt-bridge interactions are shown as black dashed lines. See also Supplementary Fig. S5.

A structure-comparison search of the Protein Data Bank using the *DALI* server (Holm, 2020 ▸) suggested structural similarity (*Z*-score 11–12, an indicator of structural similarity) to members of the CD36 superfamily of scavenger receptor proteins, including the human lysosomal integral membrane protein 2 (LIMP-2) and CD36, a fatty-acid transporter. The overall shape of MSMEG_0317Δ has similarity to LIMP-2 and CD36, which also adopt an asymmetric β-barrel core (Fig. 3 ▸ *a*). Interestingly, the three-helix bundle atop the extended β-strands in LIMP-2 and CD36 is absent in MSMEG_0317Δ; instead, a single α-helix (α14) protrudes out from the β-barrel core. Like LIMP2 and CD36, MSMEG_0317Δ encloses central cavities that span the entire length of the molecule (Fig. 3 ▸ *b*). However, unlike CD36 (PDB entry 5lgd), no additional electron density within the cavity that corresponds to a hydrocarbon chain was detected in MSMEG_0317, despite it being expressed in its native host *M. smegmatis*.

The central cavity in MSMEG_0317Δ (cavity 1, volume 340 Å^3^) adopts an uneven shape and is lined by several hydrophobic as well as charged residues (Table 2 ▸, Fig. 3 ▸ *b*, Supplementary Fig. S5). Cavity 1 has two openings: entrance 1 and entrance 2 (Figs. 3 ▸ *a* and 3 ▸ *b*). Entrance 1, which is predicted to be located close to the membrane in the native protein, has an opening of ∼8 Å (distance measured between the side chains of Glu314 and Thr45 and between Ala310 and Ile43) and is lined by Gln307, Ala310 and Glu314 of α14, Arg163 of loop 6, Ile43 of loop 1 and Thr45 of β1 (Fig. 3 ▸ *c*). Interestingly, Glu314 of α14 forms a salt-bridge interaction with Arg163 of loop 6 and this interaction is likely to contribute to the narrow opening of this cavity and holds the α14 helix in its conformation protruding out of the β-barrel core. Entrance 2 of cavity 1 has a wider opening of ∼10 Å (distance measured between the side chains of Gln181 and Leu114 and between Leu155 and Asp227) and is surrounded by Leu114 and Asp116 of β5, His157 and Leu155 of loop 6, Asp226 and Tyr224 of loop 9 and Gln181 of β7 (Fig. 3 ▸ *d*). This entrance is in the vicinity of the disordered region of loop 6 (129–154), which is likely to affect the opening and closing of this entrance. In addition to the central cavity, there is an additional smaller cavity (cavity 2, volume 41 Å^3^) at the base of the barrel surrounded by the two α-turns α2 and α10, strands β9 and β11 and the tip of β1, β3, β12 and β13 (Fig. 3 ▸ *e*, Table 2 ▸). Together, these two cavities span the entire length of the MSMEG_0317Δ molecule.

### 3.5. Xenon derivatization of the MSMEG_0317Δ crystal reveals conformational flexibility

**Figure 4:**
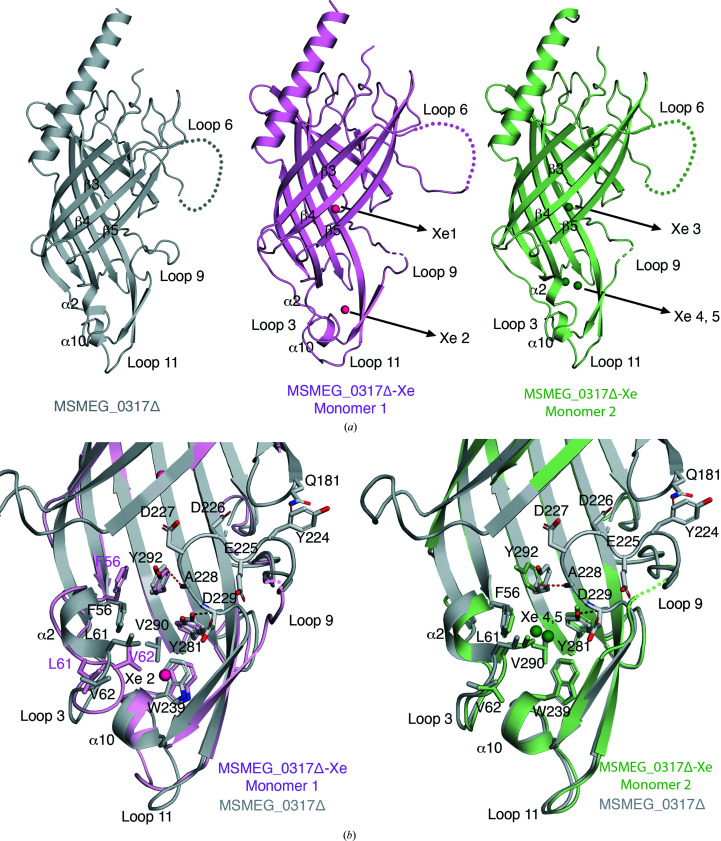
The crystal structure of xenon-derivatized MSMEG_0317Δ (referred to as MSMEG_0317Δ-Xe). (*a*) Comparison of the crystal structures of monomer 1 and monomer 2 of MSMEG_0317Δ with MSMEG_0317Δ-Xe. The positions of the five Xe atoms (Xe 1 to Xe 5) in monomers 1 and 2 of MSMEG_0317Δ-Xe are highlighted. The disordered loops 6 and 9 are shown as dotted lines. (*b*) Overlay of the crystal structure of MSMEG_0317Δ with MSMEG_0317Δ-Xe and close-up view of the base of the β-barrel core to highlight conformational flexibility near the region of loop 3, the α2 turn, loop 11 and loop 9. Loop 9 adopts a closed conformation in MSMEG_0317Δ, while in MSMEG_0317Δ-Xe loop 9 is disordered (dotted line). Hydrogen-bond interactions are shown as black dashed lines and van der Waals interactions are shown as red dashed lines. See also Supplementary Fig. S6.

To gain further insight into the potential roles of the enclosed cavities in MSMEG_0317Δ and investigate their hydrophobicity and potential to binds lipids, we pressurized MSMEG_0317Δ crystals in a xenon pressure cell (Australian Synchrotron) before cryocooling, following established protocols (Panjikar & Tucker, 2002 ▸). Xenon is known to rapidly diffuse into hydrophobic pockets of proteins with high occupancy, which permits structure determination and the identification of hydrophobic channels (Schiltz *et al.*, 2003 ▸). We solved the xenon-pressurized MSMEG_0317Δ crystal structure to 1.8 Å resolution in space group *P*1 (MSMEG_0317Δ-Xe; Table 1 ▸). Overall, the conformation of MSMEG_0317Δ-Xe is very similar to the original crystal structure, with two molecules in the asymmetric unit (monomer 1, root-mean-square deviation of 0.399 Å over 199 C^α^ atoms; monomer 2, root-mean-square deviation of 0.215 Å over 204 C^α^ atoms). However, changes in β3, β4 and β5 were noted: strand β3 was shorter and more flexible in the MSMEG_0317Δ-Xe structures, while the β4 and β5 strands were longer (Fig. 4 ▸ *a*) than in the original MSMEG_0317Δ crystal structure. Loop 6 was disordered (residues 129–151 in monomer 1 and residues 129–154 in monomer 2), as previously observed. A total of five Xe sites [anomalous peaks Xe 1 (6.3σ), Xe 2 (9.6σ), Xe 3 (10.9σ), Xe 4 (8.4σ) and Xe 5 (8.4σ)] were identified within the two monomers of the asymmetric unit and were refined (Fig. 4 ▸ *a*, Supplementary Fig. S6*a* ). Of these, Xe 1 and Xe 3 occupied an identical position in the central cavity within the two monomers. However, while the three remaining xenon sites identified were all located within cavity 2 at the base of the β-barrel core, their exact positions within the cavity differ (Xe 2, Xe 4 and Xe 5; Supplementary Fig. S6*b* ). Importantly, the binding of Xe atoms to cavity 2 (Xe 2, Xe 4 and Xe 5) resulted in a notable conformational change in loop 9 (residues 222–229). Consequently, a distinctly charged motif within loop 9 (E_225_DDAD_229_) is disordered in both monomers (Fig. 4 ▸ *b*). In our MSMEG_0317Δ crystal structure, the electron density of loop 9 is well resolved except for the side chains of Asp226 and Asp227. Loop 9 in this conformation is stabilized by a number of van der Waals interactions, including those of Tyr224 in loop 9 with Gln181 in β7 and of Ala228 in loop 9 with Tyr292 in β14, and a hydrogen-bond interaction between the main chain of Asp229 in loop 9 and the hydroxyl group of Tyr281 in β12. Xenon pressurization led to the opening of cavity 2 and destabilization of these interactions, resulting in a disordered loop 9 (Fig. 4 ▸ *b*). Additionally, the different positions of the Xe atoms in this region between the two monomers result in slightly different conformations of loop 3, loop 11 and the α2 turn, especially residues Phe56, Leu61 and Val62 (Fig. 4 ▸ *b*). Overall, xenon binding revealed plasticity of loop 9 and its surrounding region and indicated that loop 9 may adopt alternate conformations depending on ligand binding. In contrast to cavity 2, the binding of Xe atoms within cavity 1 did not result in changes in the side-chain conformation of the residues surrounding the xenon, with the exception of Leu114, and did not significantly increase the volume of cavity 1 (Supplementary Fig. S6*c* ). A longer incubation time in the pressure chamber did not result in additional xenon sites, suggesting that most of the conformational flexibility due to xenon binding occurs near the base of the β-barrel core and especially in the conformation of loop 9.

### 3.6. *AlphaFold*2-predicted structures of MSMEG_0317 and *M. tuberculosis* Rv0227c support conformational flexibility

**Figure 5:**
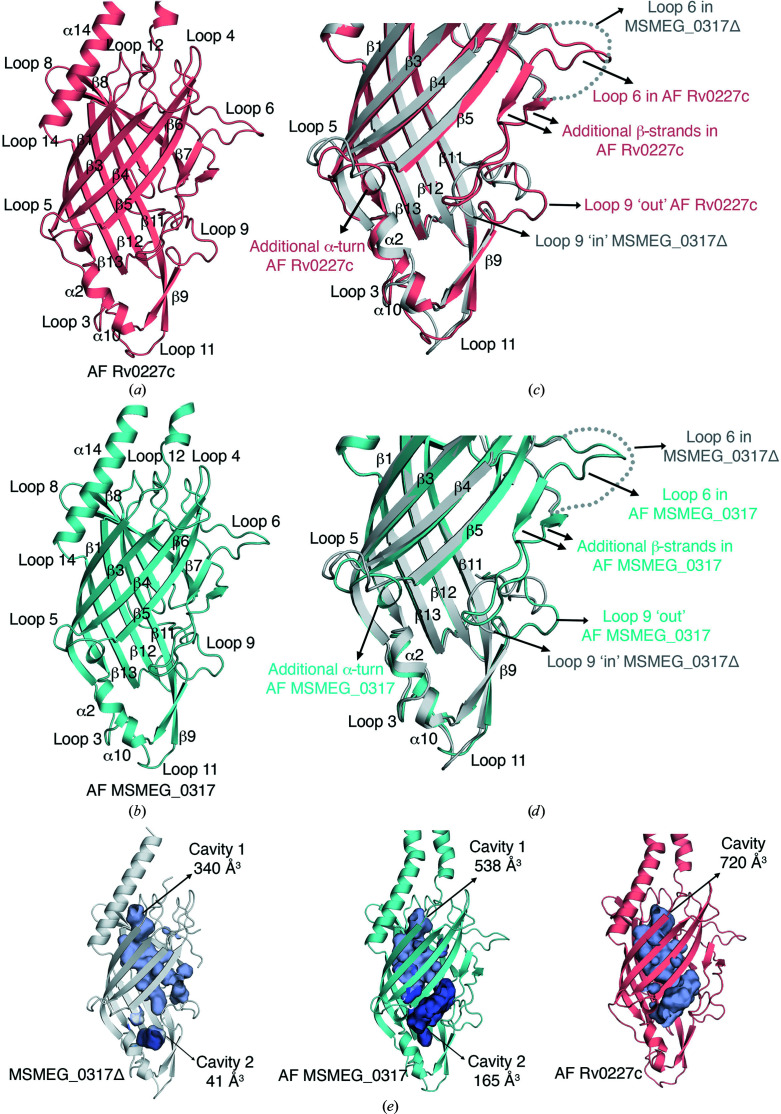
*AlphaFold*2-derived predictions of MSMEG_0317 (AF MSMEG_0317) and Rv0227c (AF Rv0227c). (*a*) Structure of AF Rv0227c. (*b*) Structure of AF MSMEG_0317Δ. (*c*) Overlay of the crystal structure of MSMEG_0317Δ with AF Rv0227c and a close-up view of the base of the β-barrel core. Loop 6 in AF Rv0227c is resolved and this loop contains two additional β-strands. Loop 9 in AF Rv0227c adopts an ‘out’ or ‘open’ conformation, in contrast to loop 9 in the MSMEG_0317Δ crystal structure, which adopts an ‘in’ or ‘closed’ conformation. (*d*) Overlay of the crystal structure of MSMEG_0317Δ with AF MSMEG_0317 and a close-up view of the base of the β-barrel core. Loop 6 in AF MSMEG_0317 is resolved in a similar position as in AF Rv0227c, including the additional two β-strands. Like AF Rv0227c, loop 9 in AF MSMEG_0317 adopts an ‘out’ or ‘open’ conformation. (*e*) Comparison of the enclosed cavities of the MSMEG_0317Δ, AF Rv0227c and AF MSMEG_0317 models. See also Supplementary Fig. S7.

While this manuscript was in preparation, the AlphaFold Protein Structure Database became available (Jumper *et al.*, 2021 ▸), enabling the prediction of three-dimensional protein structures from the human proteome and 20 other organisms, including *M. tuberculosis*. We therefore used *AlphaFold*2 to predict the three-dimensional structure of *M. tuberculosis* Rv0227c (UniProt P96409), the closest MSMEG_0317 homolog (Supplementary Fig. S1). *AlphaFold*2 predicted Rv0227c to be a ‘probable conserved membrane protein’, with most of the structure having a very high (>90) per-residue confidence score (pLDDT; Fig. 5 ▸ *a*; Supplementary Fig. S7*a* ). The predicted *AlphaFold*2 structure of Rv0227c (referred to as AF Rv0227c) is very similar to the MSMEG_0317Δ crystal structure (root-mean-square deviation of 0.618 Å over 210 C^α^ atoms), suggesting a high 3D structural similarity (Fig. 5 ▸ *a*, Supplementary Fig. S7), despite the two proteins displaying 65% sequence identity (Supplementary Fig. S1). Importantly, the predicted *AlphaFold*2 structure of MSMEG_0317 (referred to as AF MSMEG_0317) is very similar to our MSMEG_0317Δ crystal structure (root-mean-square deviation of 0.546 Å over 210 C^α^ atoms), further validating our experimental structural data (Fig. 5 ▸ *b*, Supplementary Fig. S7*b* ).

Both the AF Rv0227c and AF MSMEG_0317 models adopt an elongated β-barrel core (Figs. 5 ▸ *a* and 5 ▸ *b*; Supplementary Fig. S7). However, in addition to the 11 β-strands seen in the MSMEG_0317Δ crystal structure, the *AlphaFold*2-predicted models have two additional short β-strands within loop 6 (Figs. 5 ▸ *c* and 5 ▸ *d*); loop 6 is fully resolved in the models, but with a lower pLDDT score (70–90) in this region. In our MSMEG_0317Δ structure, loop 6 (residues 129–154) is disordered and would clash with the symmetry-related molecule if it were to adopt the conformation seen in the *AlphaFold*2 models (Figs. 2 ▸, 5 ▸ *c* and 5 ▸ *d*). This suggests that loop 6 is likely to adopt alternate conformations, as suggested by the lower pLDDT score. Moreover, the *AlphaFold*2 models reveal a third α-turn in loop 5, in addition to the two α-turns seen in our experimental MSMEG_0317Δ structure, however with a lower pLDDT score (70–90), again indicative of flexibility (Figs. 5 ▸ *c* and 5 ▸ *d*). Among the other loops connecting the secondary structures, the conformations of loops 2, 3, 4, 7, 8, 10, 12, 13 and 14 in the *AlphaFold*2 models are almost identical to those in the MSMEG_0317Δ crystal structure, while the conformations of loops 5, 9 and 11 vary (Figs. 5 ▸ *c* and 5 ▸ *d*). Of these loops, the conformation of loop 9, which is located at the base of the β-barrel core (cavity 2), deviates the most from our experimental crystal structure and adopts a more ‘open’ or ‘out’ conformation compared with the ‘closed’ or ‘in’ conformation seen in the MSMEG_0317Δ crystal structure (Figs. 5 ▸ *c* and 5 ▸ *d*). In the AF MSMEG_0317 model, the conformation of loop 9 in the ‘open’ conformation is stabilized by van der Waals interactions between Val138 and Pro141 in loop 6 and Leu223 and Tyr 224 in loop 9 (also conserved in AF Rv0227c) and a salt-bridge interaction of Lys140 in loop 6 with Glu225 in loop 9 (not conserved in AF Rv0227c, where lysine is replaced by an alanine) (Supplementary Fig. S7*d* ). While loop 6 is disordered in the MSMEG_0317Δ and MSMEG_0317Δ-Xe crystal structures, direct comparison of the loop 9 conformation in MSMEG_0317Δ (closed conformation), MSMEG_0317Δ-Xe (disordered) and the *AlphaFold*2-predicted models (open conformation) suggests that loops 9 and 6 may have an interdependent role in opening or closing of the cavity. It is thus likely that when loop 6 adopts the conformation seen in the *AlphaFold*2-predicted models, loop 9 is in an ‘open’ conformation. Together, our experimentally derived data and the *AlphaFold*2 models support the hypothesis that the solved crystal structure of MSMEG_0317Δ represents a ‘closed’ conformation.

### 3.7. Loop conformational flexibility and cavity size

We next analysed the impact of the conformations of loops 6 and 9 on the size of the enclosed cavity. A *CASTp* analysis of our crystal structure highlighted two separate cavities: cavity 1 (340 Å^3^) and cavity 2 (41 Å^3^) (Fig. 3 ▸ *b*). Interestingly, in the AF MSMEG_0317 model, the ordered conformation of loop 6 combined with the ‘out’ conformation of loop 9 result in an increase in the size of these two cavities (cavity 1, 538 Å^3^; cavity 2, 165 Å^3^; Fig. 5 ▸ *e*). Interestingly, *CASTp* analysis of the Rv0227c *AlphaFold* model predicted a single, large cavity occupying the entire length of the molecule (720 Å^3^; Fig. 5 ▸ *e*). Despite the relatively high conservation of the residues surrounding the cavities between MSMEG_0317Δ and Rv0227c (Table 2 ▸, Supplementary Fig. S1), the sequence differences between Rv0227c and MSMEG_0317, and the differences in the conformations of the loops, especially loops 6 and 9, and the conformation of the α14 helix are likely to influence the shape and the volume of these cavities (Fig. 5 ▸). While xenon derivatization of crystals did not clearly identify a hydrophobic channel and further work will be required to identify the native ligand that directly binds to MSMEG_0317Δ, xenon derivatization and analysis of the *AlphaFold*2 models has enabled the identification of elements that may allow ‘open’ or ‘closed’ conformations in MSMEG_0317Δ.

### 3.8. The β-barrel fold is predicted to be conserved in *C. glutamicum* NCgl2760

**Figure 6:**
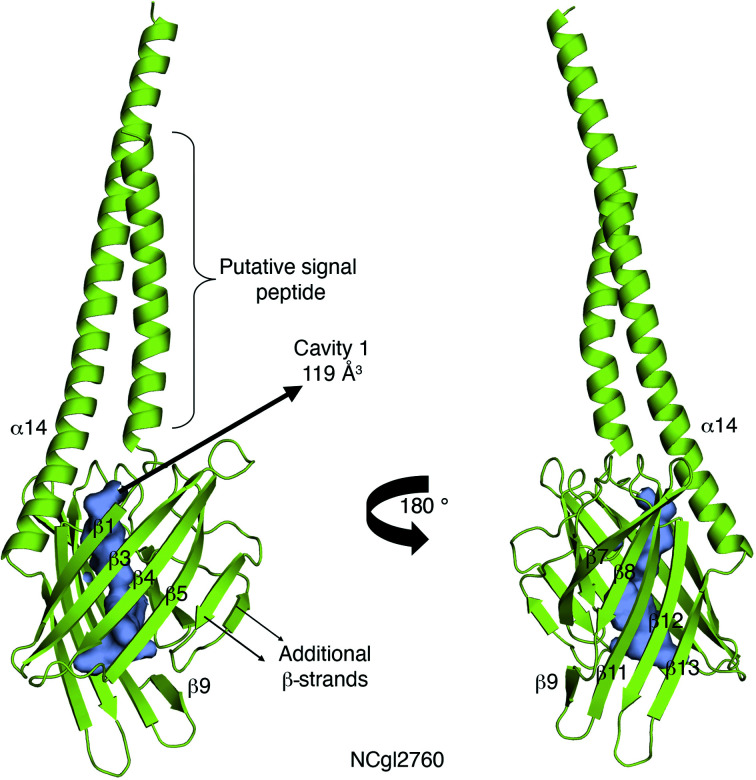
*AlphaFold*2-derived prediction of NCgl2760 (AF NCgl2760). AF NCgl2760 adopts a smaller β-­barrel core compared with MSMEG_0317 and Rv0227c; however, the central cavity is still a conserved feature. Note that the N-terminal helix may represent a signal peptide. See also Supplementary Figs. S8 and S9.

*C. glutamicum* NCgl2760 is the best match for MSMEG_0317 in the *C. glutamicum* proteome, with the proteins sharing 24% amino-acid sequence identity (Supplementary Fig. S1). Despite the modest sequence identity, both proteins are encoded by the same well conserved cell-wall biosynthesis locus (Fig. 1 ▸ *b*), providing further evidence that they are orthologs. To understand the structural basis of this conservation, we next used *AlphaFold*2 to generate a model of NCgl2760 (Fig. 6 ▸). The root-mean-square deviation of NCgl2760 with MSMEG_0317 is 1.401 Å over 98 C^α^ atoms. The NCgl2760 model adopts a much smaller β-barrel core, with 12 β-strands, compared with the extended β-barrel core seen in MSMEG_0317 and Rv0227c. Despite this, the positions of strands β1, β3, β4, β5, β7, β8, β11, β12 and β13 in MSMEG_0317Δ align with β-strands in NCgl2760, with the exception of the β9 strand (Supplementary Fig. S8). The β9 strand, which is connected to the β8 strand through loop 9, is located at the base of the β-barrel core in MSMEG_0317Δ and Rv0227c. In contrast, in NCgl2760 loop 9 is much shorter and the β9 strand is part of the main β-barrel core. Interestingly, the two additional β-strands seen in loop 6 in the *AlphaFold*2 models of Rv0227c and MSMEG_0317 are also present in NCgl2760 (Fig. 6 ▸). Like MSMEG_0317Δ and Rv0227c, NCgl2760 encloses a central cavity, albeit with a different shape and volume (119 Å^3^; Fig. 6 ▸; Supplementary Fig. S9).

### 3.9. LmcA structures suggest potential functions in cell-wall lipoglycan synthesis in Corynebacterineae

A role for LmcA in cell-wall synthesis was initially proposed based on the phenotypic characterization of an *NCgl2760* null mutant of *C. glutamicum*. This strain lacks all full-length LM and LAM lipoglycans and accumulates a truncated LM species, a phenotype that is mirrored by an *mptA* mutant lacking a key mannosyltransferase responsible for synthesizing the mannan backbone (Cashmore *et al.*, 2017 ▸). The putative orthologs of NCgl2760 in mycobacteria (Rv0227c and MSMEG_0317) are essential for bacterial growth (Cashmore *et al.*, 2017 ▸; Sassetti *et al.*, 2003 ▸), hampering their characterization. While several theoretical functions of LmcA could explain the *NCgl2760* mutant phenotype, our structural characterization of the LmcA family points to a possible lipid-binding function for these proteins. Specifically, the structural similarity between MSMEG_0317Δ and CD36 with palmitate bound in a central cavity raises the possibility that the LmcA family may also bind palmitate or a lipid of similar chain length. Despite significant heterogeneity (Klatt *et al.*, 2018 ▸), the lipid core of all PIM/LM/LAM species contains at least one palmitate, and the most abundant species contain two palmitate chains (for example AcPIM2). Unlike mycobacteria, *C. glutamicum* synthesizes a second class of lipoglycans termed Cg-LM-B, which are structurally related to PIM/LM/LAM but are instead based on an α-d-glucopyranosyluronic acid-(1–3)-glycerol anchor (Lea-Smith *et al.*, 2008 ▸; Tatituri, Illarionov *et al.*, 2007 ▸; Tatituri, Alderwick *et al.*, 2007 ▸). These anchors comprise two palmitate chains, and synthesis of Cg-LM-B lipoglycans is also compromised in an *NCgl2760* null mutant (Cashmore *et al.*, 2017 ▸). A requirement to accommodate two structurally different lipid anchors could explain the structural differences between NCgl2760 and the more closely related MSMEG_0317/Rv0227c proteins. To test whether palmitate can bind to MSMEG_0317, we attempted to crystallize MSMEG_0317Δ in the presence of lipids such as palmitic acid (C16 carbon chain composition), phosphatidyl­glycerol (C8 carbon chain composition) and phosphatidyl­inositol (C8 carbon chain composition). While these crystals diffracted to high resolution, no additional electron density corresponding to the lipids was observed, consistent with the notion that the structure obtained may represent a ‘closed’ conformation and structural change may be required to allow lipid binding. An alternative hypothesis is that the LmcA family binds the mannose donor for LM/LAM biosynthesis, polyprenylphosphomannose; however, its lipid component is structurally distinct from palmitate. Overall, we speculate that lipoglycan-bound LmcA may interact with the MptA mannosyltransferase to catalyse the synthesis of the mannan backbone of LM, but further experiments are required to identify the true ligand of LmcA and investigate its inter­actions with other proteins of the LM/LAM pathway.

## 4. Concluding remarks

Here, we report the first crystal structure of the *M. smegmatis* ortholog of LmcA, MSMEG_0317, at 1.8 Å resolution. The crystal structure of the periplasmic domain of MSMEG_0317 revealed an elongated β-barrel fold which encloses two distinct cavities. The availability of *AlphaFold*2 has allowed us to directly compare our experimental MSMEG_0317Δ crystal structure with *AlphaFold*2-derived models of putative LmcA orthologs from *M. tuberculosis* (Rv0227c) and *C. glutamicum* (NCgl2760). Our study revealed three key structural features. Firstly, we identified that all three LmcA proteins adopt a β-barrel fold. In MSMEG_0317 and Rv0227c, which share 65% sequence identity, the β-barrel core adopted an elongated fold, while in NCgl2760, which shares 24% sequence identity with MSMEG_0317, the β-barrel core was significantly smaller. Secondly, by comparing our crystal structure with *AlphaFold*2-derived models of Rv0227c and NCgl2760 we have shown that the central cavity enclosed by the β-barrel fold is a common feature of the LmcA family. Thirdly, through xenon derivatization of the MSMEG_0317 crystal structure we have identified structural elements within the β-barrel that show conformational flexibility, allowing ‘open’ or ‘closed’ conformations that may drive access to the enclosed cavities. Further work will be required to identify the authentic ligand that binds to the LmcA family; however, the observed structural features suggest a lipid-binding function for LmcA and provide clues to the flexible regions where conformational changes may occur upon ligand binding.

## Supplementary Material

PDB reference: MSMEG_0317Δ, 7n3v

PDB reference: xenon derivative, 7shw

Supplementary Figures. DOI: 10.1107/S2059798322001772/jb5039sup1.pdf

## Figures and Tables

**Table 1:** Data-collection, phasing and refinement statistics Values in parentheses are for the highest resolution shell.

	MSMEG_0317Δ old native data	MSMEG_0317Δ new native data	MSMEG_0317Δ-KI (merged)	MSMEG_0317-Xe
Data collection				
Space group	*P*1	*P*1	*P*1	*P*1
*a*, *b*, *c* (Å)	34.50, 57.44, 73.99	34.54, 57.54, 73.81	34.40, 57.67, 73.58	34.12, 56.99, 73.57
α, β, γ (°)	102.93, 90.09, 99.92	102.57, 90.17, 100.23	102.65, 90.00, 100.26	78.42, 89.21, 78.92
Wavelength (Å)	0.9537	0.9537	1.4586	0.9537
Resolution (Å)	39.63–1.99 (2.05–1.99)	39.76–1.83 (1.87–1.83)	39.76–2.43 (2.52–2.43)	48.58–1.79 (1.83–1.79)
Total reflections	135294	277229	169742	357545
Unique reflections	35855	46866	19703	48586
*R*_merge_ (%)	9.0 (28.8)	7.4 (52.3)	3.3 (9.7)	8.7 (4.8)
〈*I*/σ(*I*)〉	9.9 (4.1)	12.1 (2.9)	50 (21.7)	12.4 (2.4)
Completeness (%)	96.5 (93.1)	97.1 (86.0)	96.4 (94.0)	93.5 (76.5)
Multiplicity	3.8 (3.7)	5.9 (5.5)	8.6 (8.4)	7.4 (3.7)
CC_1/2_	0.988 (0.901)	0.998 (0.762)	0.999 (0.995)	0.998 (0.755)
CC_ano_	—	—	0.557 (0.201)	−0.124 (−0.070)
Refinement				
Resolution (Å)		18.45–1.83		48.58–1.79
*B* factors (Å^2^)				
Overall		30.4		30.3
Protein		29.6		29.8
Water		39.8		39.7
Xenon		—		41.2
*R*_work_/*R*_free_ (%)		18.0/21.3		19.7/21.0
No. of atoms				
Protein		4123		3976
Other		16		6
Water		330		202
Xenon		—		5
Ramachandran plot				
Favoured (%)		97.7		98
Allowed (%)		2.3		2
R.m.s. deviations				
Bond lengths (Å)		0.009		0.006
Bond angles (°)		0.973		0.811
*MolProbity* score		0.86		0.85

**Table 2:** Residues surrounding the cavity in MSMEG_0317Δ Residues in bold denote those that are conserved in Rv0227c.

Cavities	Structural elements	Residues surrounding the cavity	Cavity volume (Å^3^)
Cavity 1			340
	β1; β3	Thr45, **Leu47**, **Ser49**; Leu76, **Gln78**, Met80	
	β4	**Val95**, Thr97, **Leu99**	
	β5; β6	**Leu112**, **Leu114**, **Asp116**; **Phe175**, **Phe176**, **Asp177**	
	β7; β8	**Gln181**; **Gln204**	
	β11	**Arg253**, **Tyr255**, **Arg259**, **Phe261**	
	β12	Ser273, **Glu275**, Gly277, Gln279	
	β13	Tyr292, Val293, Phe295, Val297	
	α14	**Gln307**, Ala310, **Glu314**	
	Loop 1	**Ile43**	
	Loop 6	Met120, **Leu155**, **His157**, Thr161, **Tyr162**, **Arg163**	
	Loop 9	Ile218, **Tyr220**, Ser221, **Tyr224**, Asp226, **Asp227**, Ala228	
Cavity 2			41
	α2 turn	**Leu61**	
	α10 turn	**Trp239**	
	β1	Phe56	
	β3	Ile68	
	β13	**Val290**, Tyr292	
	β9	**Val232**	
	β12	**Tyr281**	
	Loop 9	Ala228, **Asp229**	
	Loop 3	Phe66	
