# Supplementary material for: Crystal structure of the putative cell-wall lipoglycan biosynthesis protein LmcA from Mycobacterium smegmatis
Source: Acta Crystallogr D Struct Biol. 2022 Mar 11;78(Pt 4):494–508. doi: 10.1107/S2059798322001772 (PMC8972800; doi:10.1107/S2059798322001772)
Supplement: Supplementary file 1 [file d-78-00494-sup1.pdf]

Acta Cryst D

STRUCTURAL
BIOLOGY

Volume 78 (2022)

Supporting information for article:

Crystal structure of the putative cell-wall lipoglycan biosynthesis protein LmcA from *Mycobacterium smegmatis*

Onisha Patel, Rajini Brammananth, Weiwen Dai, Santosh Panjikar, Ross L. Coppel, Isabelle S. Lucet and Paul K. Crellin

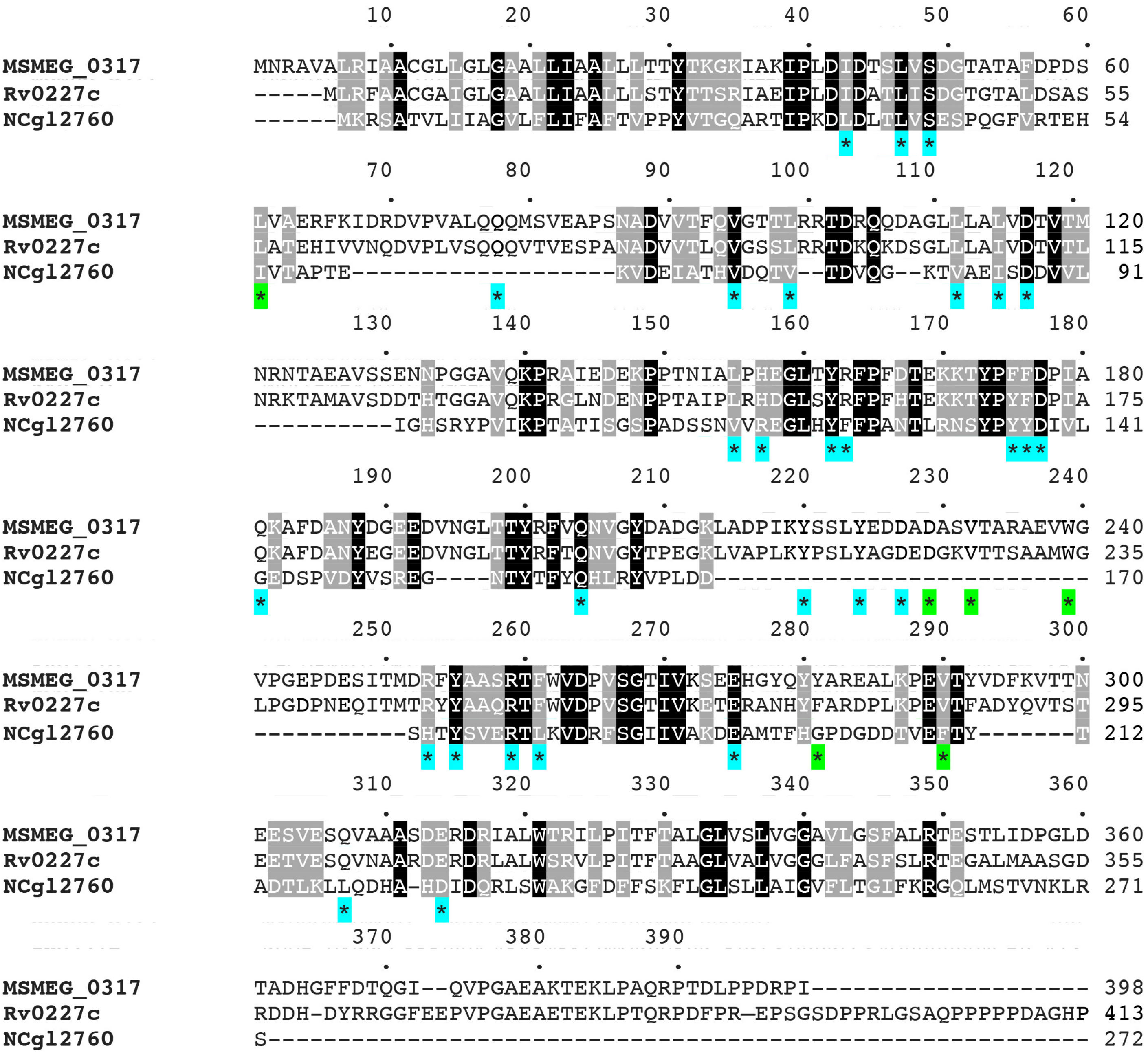

Fig. S1. Sequence alignment of *M. smegmatis* MSMEG_0317 with *M. tuberculosis* Rv0227c and C. *glutamicum* NCgl2760. MSMEG_0317 display 65% sequence identity with *M. tuberculosis* Rv0227c and 24% with NCgl2760. Identical residues are shaded black while similar residues are shaded grey. The black dots represent sequence numbering. Blue asterisks denote MSMEG_0317 residues in cavity 1 that are conserved in Rv0227c, and green asterisks denote MSMEG_0317 residues in cavity 2 that are conserved in Rv0227c.

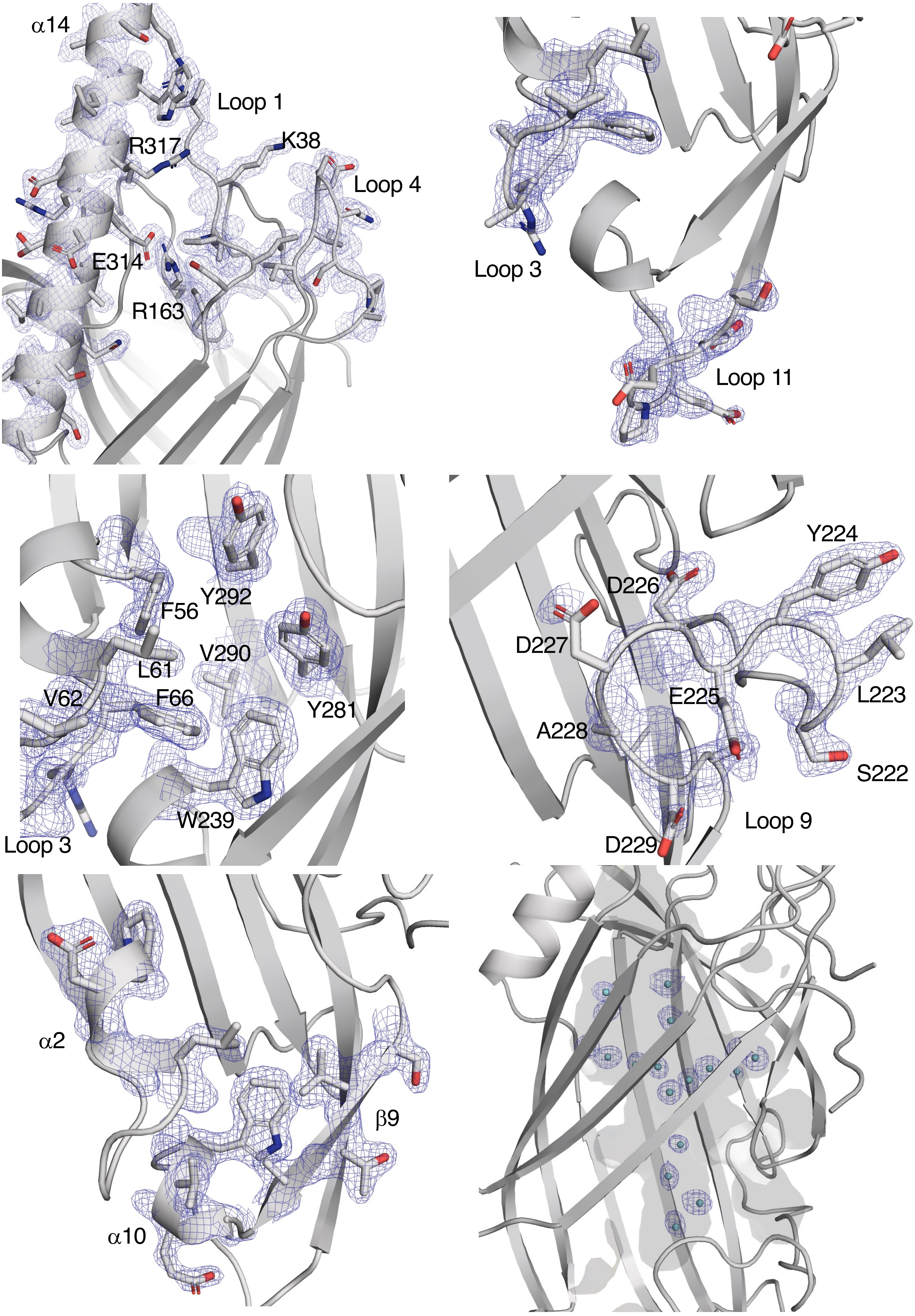

Fig. S2. 2Fo-Fc map of some amino acid side chains and water molecules (blue spheres) inside the cavity (shown in shade of grey) in the MSMEG_0317Δ crystal structure after refinement and model building contoured at 1σ.

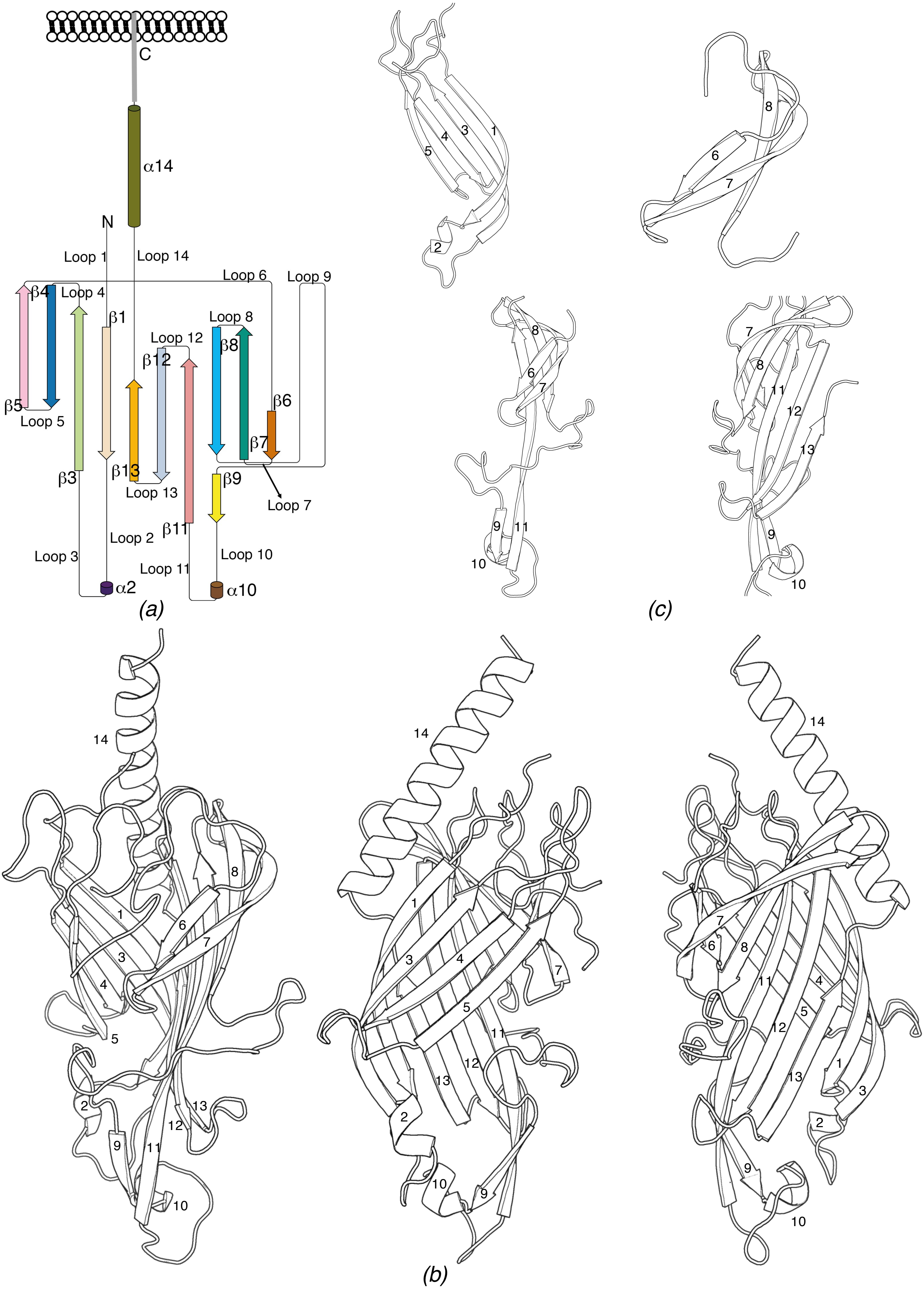

Fig. S3. (a) Topology diagram of MSMEG_0317Δ generated using Pro-origami. (b) A black and white trace of the overall structure highlighting secondary elements (shown in numbers) and their arrangement. (c) A close up of secondary elements to highlight their arrangement relative to each other.

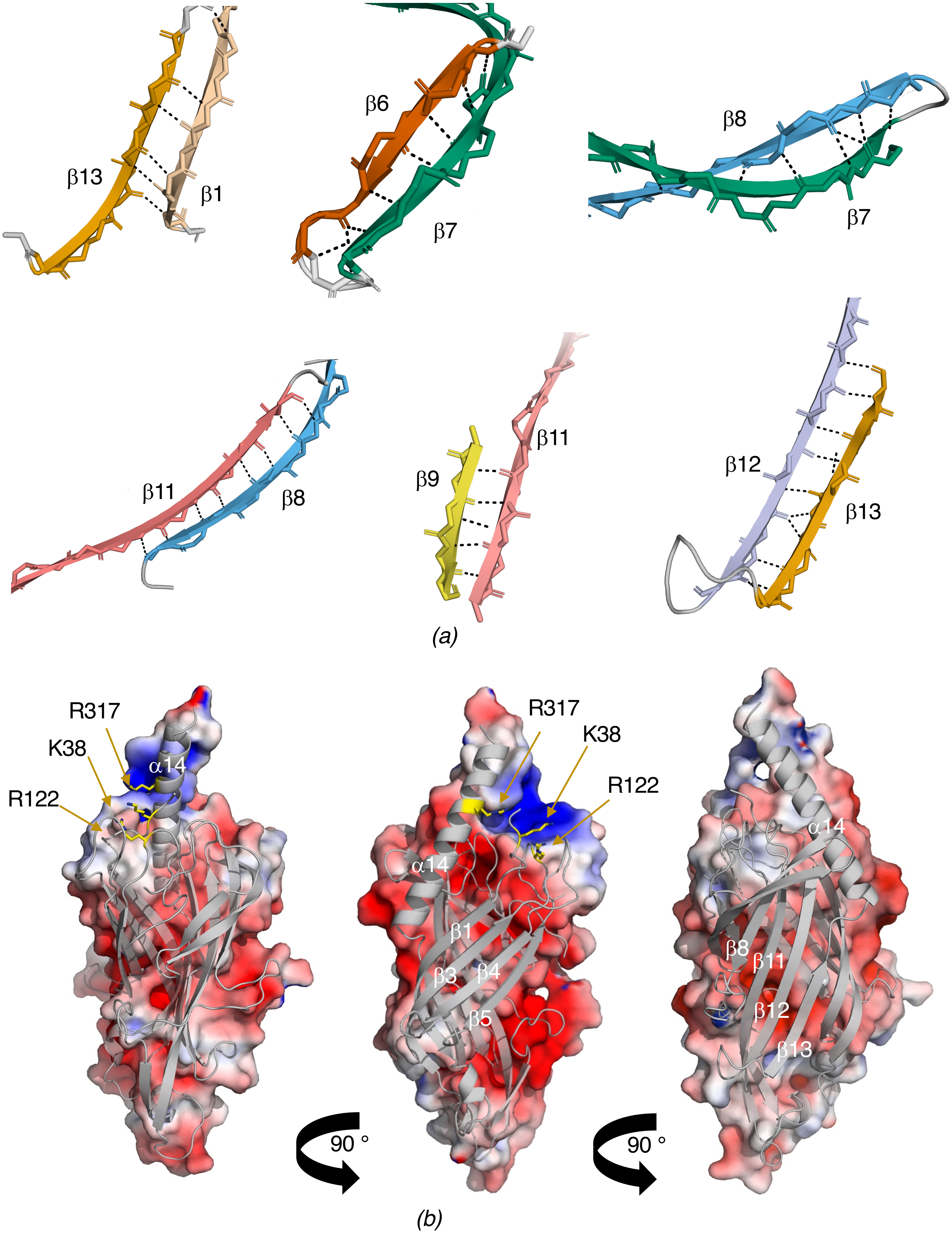

Fig. S4. (a) A close up of some beta strands to show hydrogen bond interactions (black dashed lines). (b) Electrostatic surface diagram generated using the APBS plugin in PyMOL. Positively charged residues are shown as yellow sticks.

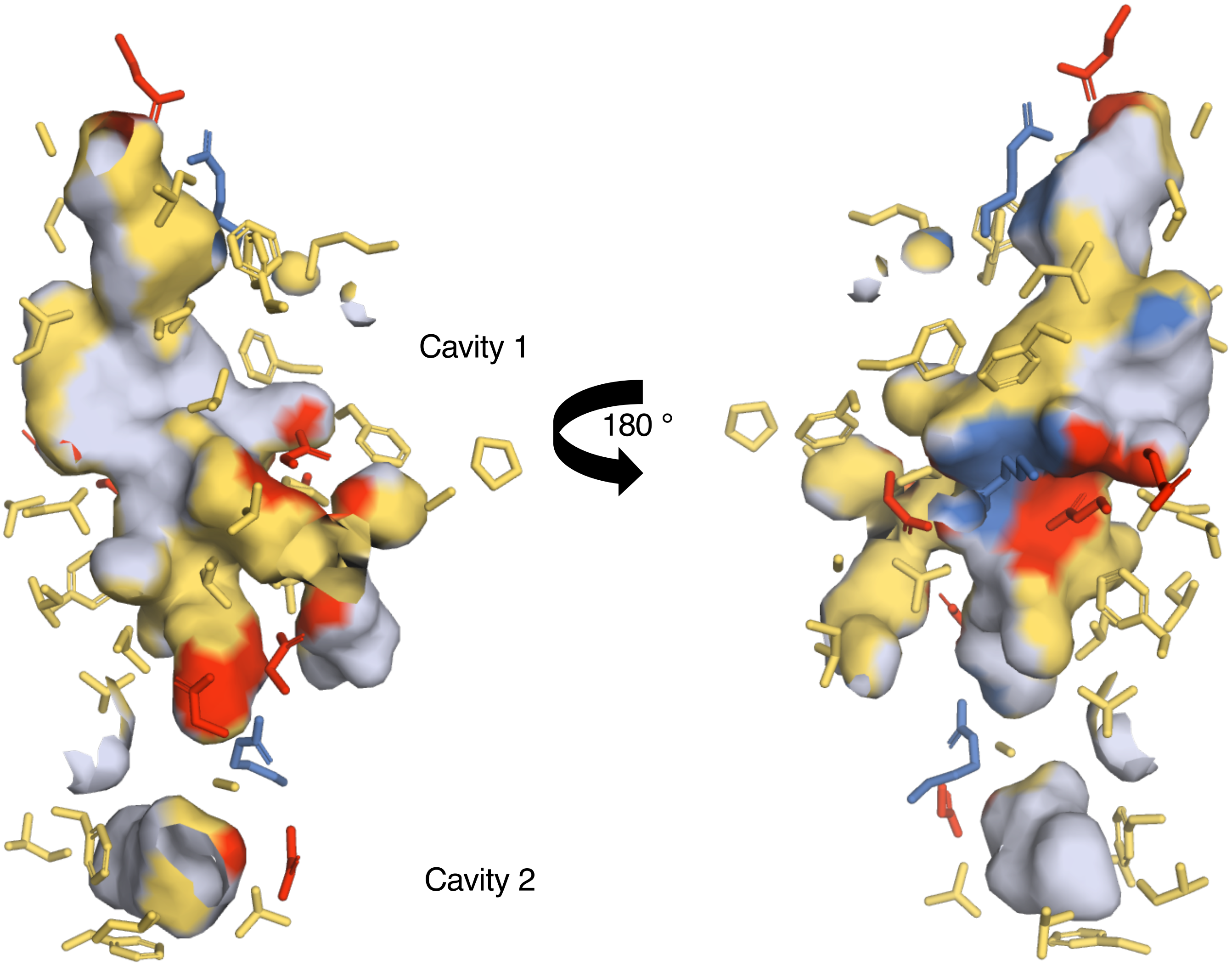

Fig. S5. (a) Residues surrounding the MSMEG_0317Δ cavities. Hydrophobic residues (Ala, Val, Leu, Ile, Pro, Phe, Gly, Met) are shown in yellow and charged residues are shown in blue (Lys, Arg) and red (Asp, Glu). The remaining surface represents polar residues (Gln, Asn, His, Ser, Thr, Tyr, Trp; residue side chains not shown in the figure for simplicity).

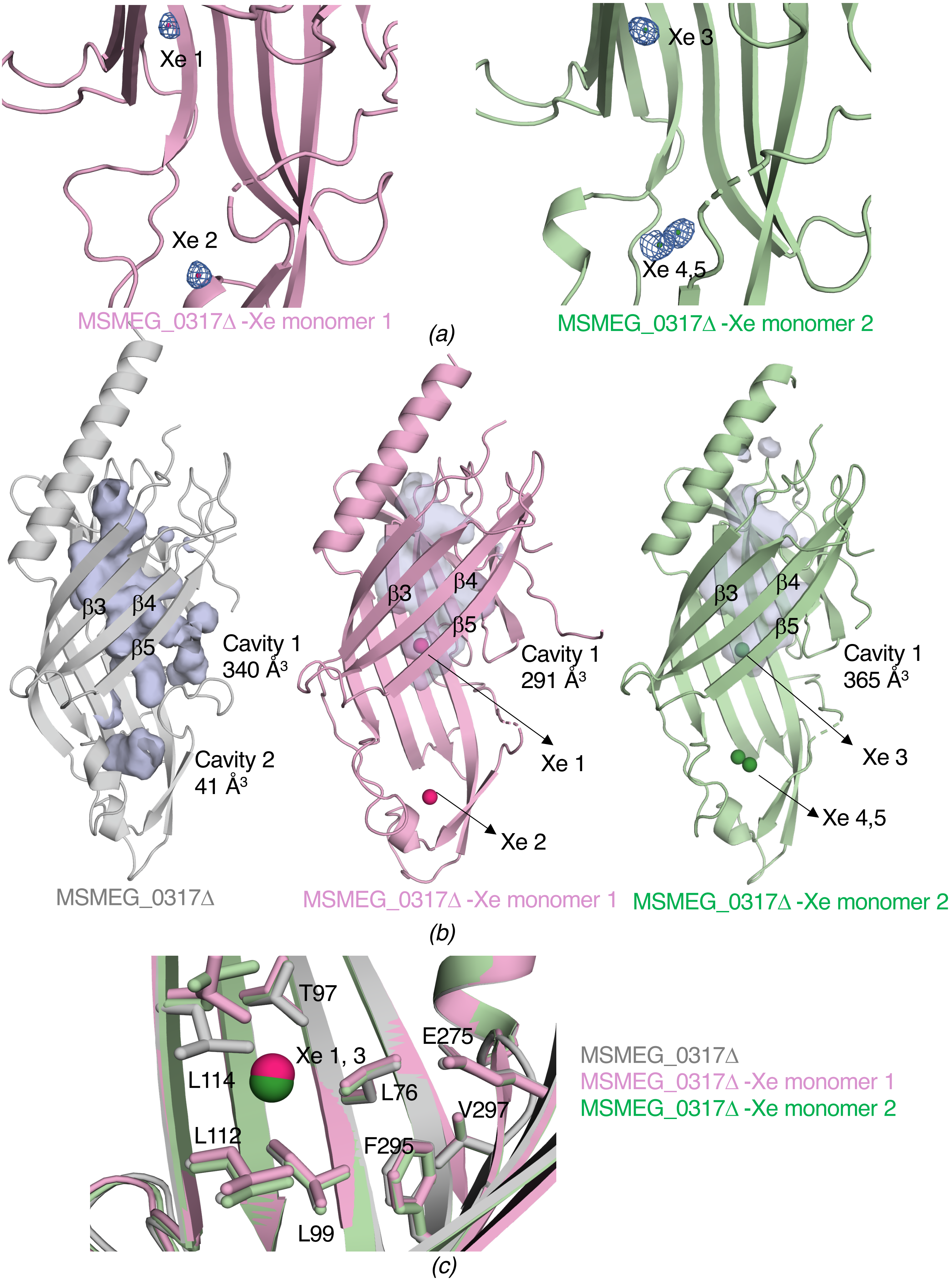

Fig. S6. (a) Anomalous electron density for xenons contoured at 3σ. (b) Cavity comparison between MSMEG_0317Δ and MSMEG_0317Δ-Xe crystal structures. Position of xenon atoms is highlighted. (c) Overlay of monomer 1 and monomer 2 of MSMEG_0317Δ-Xe with MSMEG_0317Δ crystal structures near cavity 1.

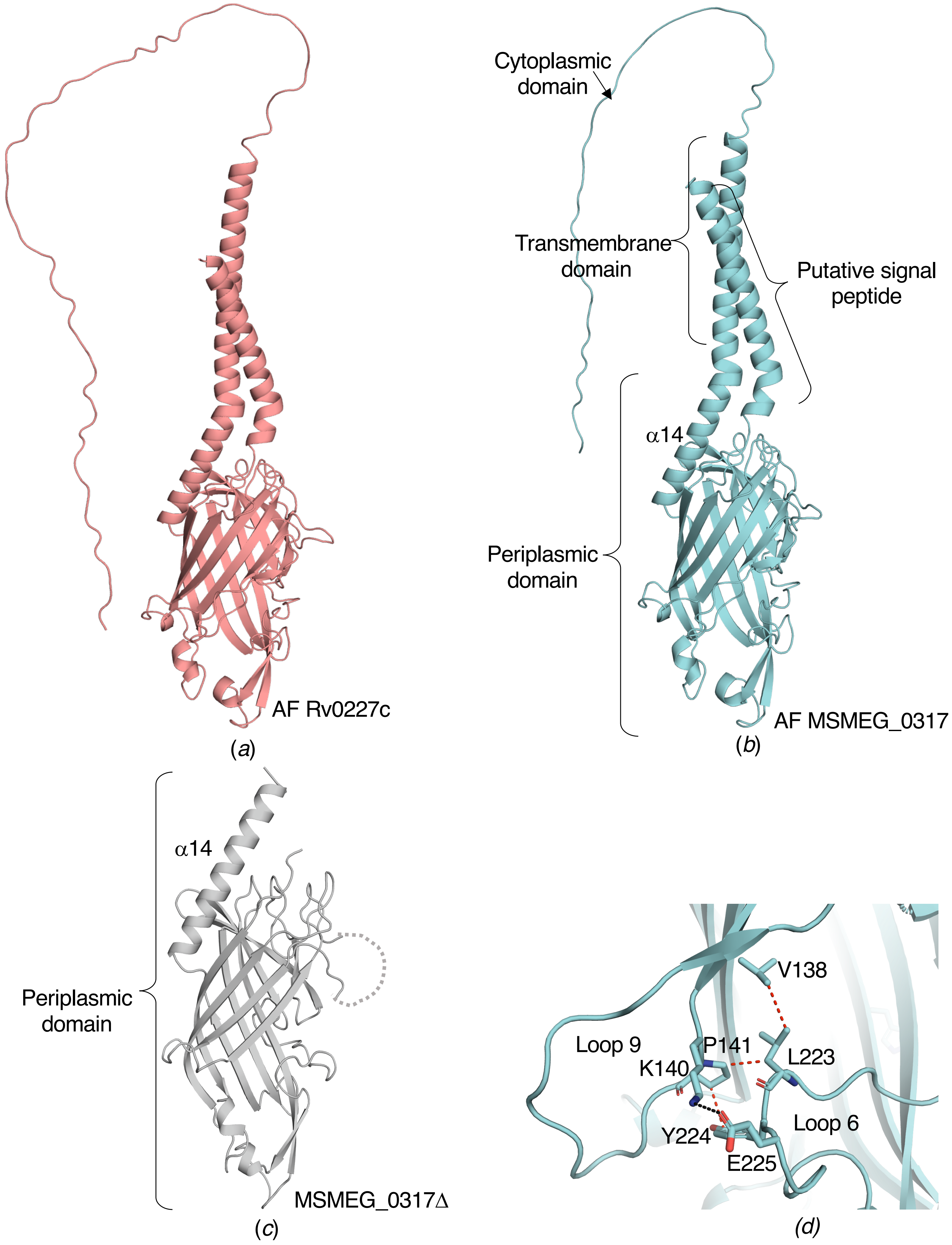

Fig. S7. (a) AlphaFold2 predicted full length structure of Rv0227c (AF Rv0227c). (b) AlphaFold2 predicted full length structure of MSMEG_0317 (AF MSMEG_0317). (c) The crystal structure of MSMEG_0317Δ. (d) Closeup view of the interaction between loop 6 and loop 9 in AF MSMEG_0317. Salt bridge interaction is shown in black dashed lines and van der waals interactions in red dashed lines.

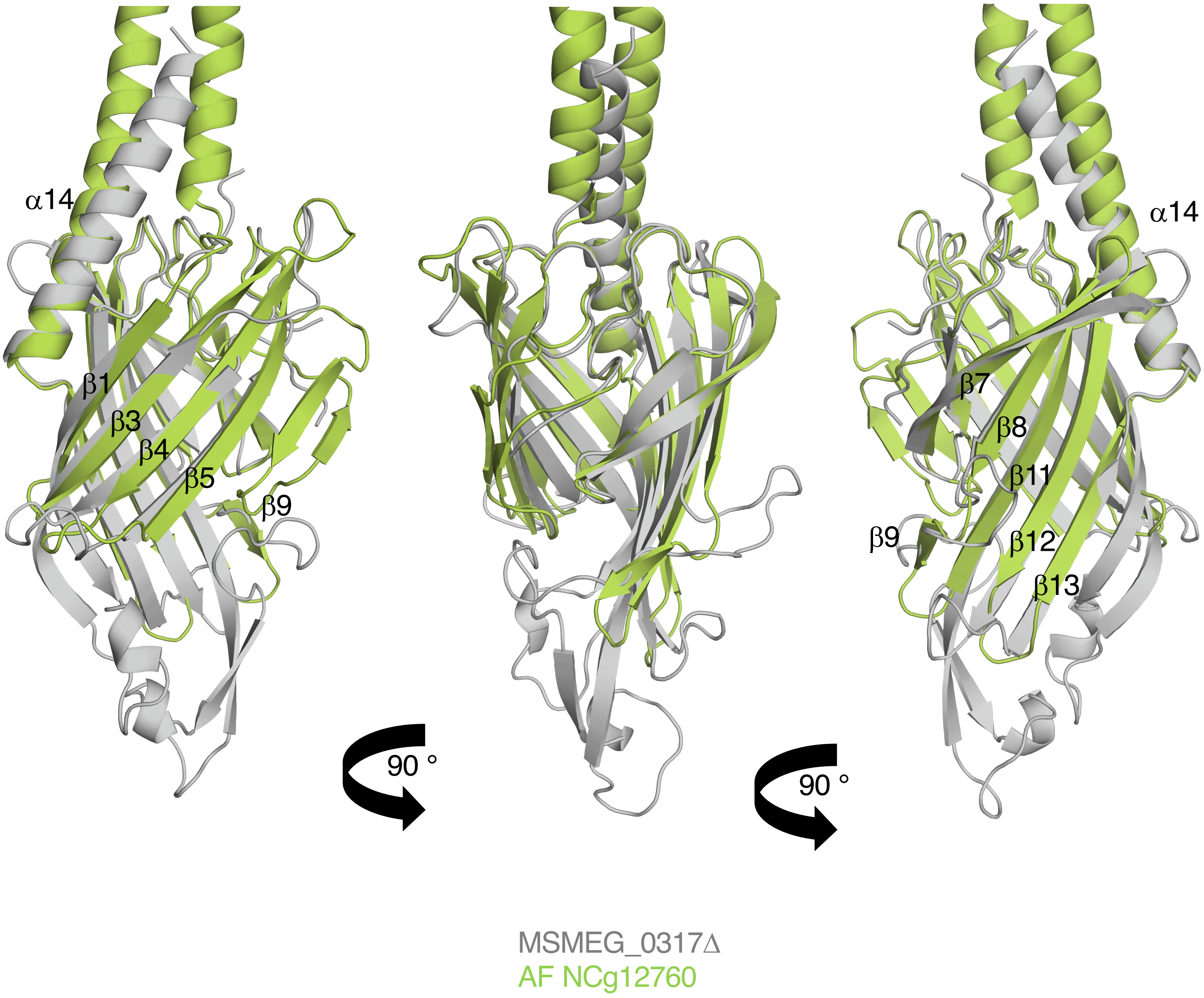

Fig. S8. Overlay of AlphaFold2 predicted structure of NCgl2760 (AF NCgl2760) with the crystal structure of MSMEG_0317Δ, showing alignment of the core β strands. The β strands numbering is based on the MSMEG_0317Δ crystal structure.

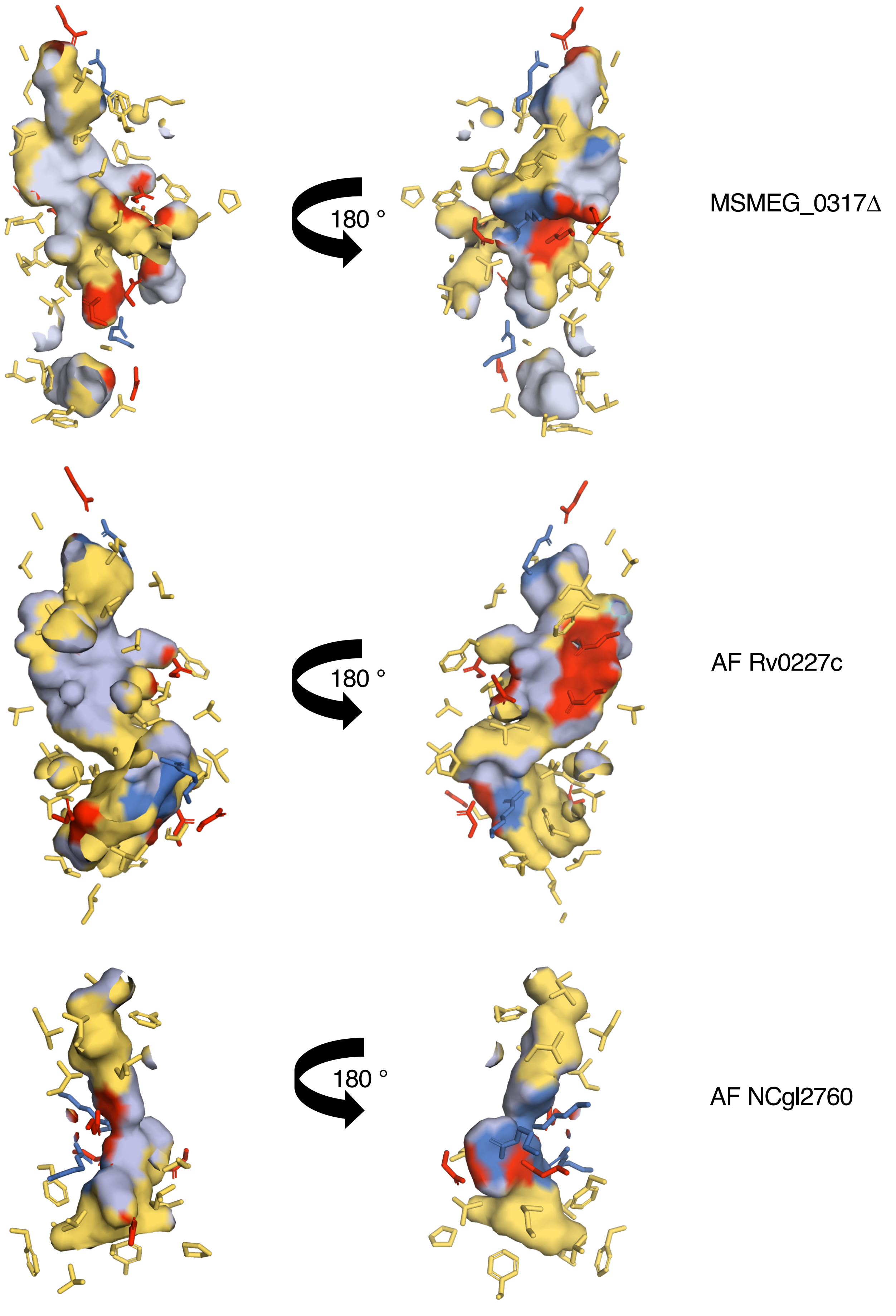

Fig. S9. Distribution of hydrophobic and charged residues surrounding the cavity in the MSMEG_0317Δ crystal structure, AlphaFold2-predicted Rv0227c (AF Rv0227c) and NCgl2760 (AF NCgl2760). Hydrophobic residues (Ala, Val, Leu, Ile, Pro, Phe, Gly, Met) are shown in yellow and charged residues are shown in blue (Lys, Arg) and red (Asp, Glu). The remaining surface represents polar residues (Gln, Asn, His, Ser, Thr, Tyr, Trp; residue side chains not shown in the figure for simplicity).
